# Analysis of Physicochemical Parameters of Congress Worts Prepared from Special Legume Seed Malts, Acquired with and without Use of Enzyme Preparations

**DOI:** 10.3390/foods10020304

**Published:** 2021-02-02

**Authors:** Alan Gasiński, Józef Błażewicz, Joanna Kawa-Rygielska, Joanna Śniegowska, Maciej Zarzecki

**Affiliations:** Department of Fermentation and Cereals Technology, Faculty of Biotechnology and Food Science, University of Environmental and Life Science, Chełmońskiego 37 Street, 51-630 Wroclaw, Poland; jozef.blazewicz@upwr.edu.pl (J.B.); joanna.kawa-rygielska@upwr.edu.pl (J.K.-R.); joanna.sniegowska@upwr.edu.pl (J.Ś.); 109619@student.upwr.edu.pl (M.Z.)

**Keywords:** malt, congress mash, legumes, vetch, pea, chickpea, lentil, antioxidant activity, brewhouse efficiency

## Abstract

This study was conducted to produce malt from legume seeds (chickpea, lentil, pea, and vetch) and test whether malting with parameters, typically barley grain, will result in well-modified legume seed malt. Analysis of malt was performed by producing congress worts from legume seed malts. Concentration of phenolic compounds, as well as antioxidant activity of legume seed malts was analysed. Acquired worts were characterised with poor technological characteristics (wort extract, wort volume, saccharification time, brewhouse efficiency); however, the malting process increased concentration of phenolic compounds and antioxidant activity of the plant material. Subsequent mashing tests with addition of different external enzymes and/or gelatinisation of legume seed malt were performed. Use of external enzymes improved saccharification time, extract content, wort volume, as well brewhouse efficiency in the case of some legume seed malts. The best brewhouse efficiencies and highest extract values were acquired by the samples prepared with 30% of gelatinised vetch malt or chickpea malt mixed with 70% of Pilsner malt. The study shows that there is possibility of creating legume seed malts, but malting and mashing characteristics need to be customised for these special malts.

## 1. Introduction

Legumes are a part of the *Leguminosae* family, which consists of 650 genera with 18,000 species. It is the third largest angiosperm family in the world. Seeds of legumes, sometimes called pulses, are the second most important source of nutrients in the world, just after cereals [1]. They are rich in carbohydrates, fats, calcium, iron, thiamine, riboflavin, and fibre, but the most important quality of legumes is their very high protein content, which can range from 16% to 50% [2]. There is increasing interest in consuming legumes, as well as other plant-based products, primarily due to three different factors: (1) awareness of climate change; (2) nutritional benefits of legume consumption; as well as (3) concerns about welfare of farm animals [3]. Unfortunately, transitioning to higher legume consumption is not as straightforward as a simple increase in legume production. Legumes are simple crops that do not need application of chemical fertilizers to maintain yield on optimal levels; moreover, growing legumes lowers the cost of production, and improves quality of the soil, due to nitrogen assimilation and carbon sequestration [4,5]. Legumes, despite their high nutritional value, are not utilised to their fullest potential because they possess few flaws, which mitigate their advantages. Legume grains are hard-to-cook, their proteins are hard to digest, the bioavailability of many minerals and vitamins present in seeds is low, and many of legumes possess antinutritional substances, such as phytic acid, tannins, trypsin inhibitors, and α-galactosides [6,7]. If it was possible to remove some or all of these substances and improve digestibility of legume protein, then legumes could possibly play a larger role in the nutrition of humankind [8]. In this research, we attempted to use the process of malting to change characteristics of seeds of some legumes grown commercially in Europe, such as vetch (*Vicia sativa*), yellow pea (*Pisum sativum*), green lentils (*Lens culinaris*), and chickpea (*Cicer arietinum*) [9]. Malting is a technological process used to modify mainly grains of barley (*Hordeum vulgare*) as well as other cereal grains, and its usefulness in modification of legume seeds is mostly unknown [10]. It is a process in which grain moisture is increased by alternating the process of submerging it in water and storing grain in humid air (in a process called “steeping”), then grains with increased moisture (up to 42–45%) are germinated and dried in ovens in a process called “kilning”. Conditions of kilning, steeping, and germination can be highly modified by maltsters (to acquire malt with different characteristics). The process of malting is used to modify the physical structure of the grains and allows for activation of many metabolic pathways in which grains change their composition, produce enzymes, and create various phytochemicals [11]. Germination of legume seeds, which is a part of the malting process, has been assessed by few research teams; studies show improvements in antioxidant ability, bioavailability of vitamins and minerals, higher concentration of polyphenols, and bioactive peptides in the germinated legume seeds [12,13,14]. These results show a possibility of using the malting process to improve the nutritional quality of legume seeds. The main advantages of malting over the sole germination process are the positive changes in the organoleptic characteristics of the finished product, as well as improved stability of malt over the germinated seed [15]. Legume malts could be used to produce gluten-free beer worts or malt extracts with high protein content, as well as to produce fermented food, such as tempeh, but with a reduced amount of antinutritional substances and an increased amount of antioxidants [2,3,8,10,11,12]. The aim of this study is to determine advantages and disadvantages of congress worts obtained from legume seed malts (chickpea, yellow pea, common vetch, and green lentil) produced in analogous malting and mashing conditions, such as congress worts made from typical Pilsen malt.

## 2. Materials and Methods

### 2.1. Reagents and Standards

Reagents used in this study were: acetic acid (99.5%), diammonium salt of 2,2′-azobis (3-ethylbenzothiazoline-6-sulfonate) (ABTS+•) (98%), 1,1-diphenyl-2-picrylhydrazil radical (DPPH•) (95%), ferrous sulphate (98%), Folin–Ciocalteu reagent (2 M), gallic acid (98%), 6-hydroxy-2,5,7,8-tetramethylchroman-2-carboxylic acid (Trolox) (97%), iron (III) oxide (99%), Lugol’s iodine (iodine and potassium iodide solution in water) (5%), methanol (99%), sodium acetate (99%), sodium carbonate (99%), and 2,4,6-tripyridyl-S-thiazine (TPTZ) (98%).

### 2.2. Legume Seeds

Legume seeds used in this study were: common vetch—VS (*Vicia sativa*), green lentil—LS (*Lens culinaris*), chickpea—CS (*Cicer arietinum*), and yellow pea—PS (*Pisum sativum*) harvested in 2019, acquired from a company supplying the food industry with seeds of various plants.

### 2.3. Malt

Malt used in this study, as a control sample, were: Pilsen malt (BM) produced by Viking Malt Company (Strzegom, Poland) from barley harvested in 2019.

### 2.4. Enzyme Preparations

Enzyme preparations used in this study were produced by DSM Food Specialties (Heerlen, The Netherlands). They are enzyme preparations with different enzymatic activities: Amigase Mega (AM: fungal amyloglucosidase, standardised activity ≥36,000 AGI/g of the preparation, batch number: 4192201011); Filtrase BR-X (FBR: thermostable β-glucanases and hemicellulases, standardised activity ≥6000 BGF/g of the preparation, batch number: 4191222011); Filtrase NL (FNL: thermostable fungal β-glucanases and xylanases, standardised activity ≥10,500 BGF/g of the preparation, batch number: 4191809011); Maxazyme NNP DS (MAX: bacterial protease, standardised activity ≥180,000 PC/g of the preparation, batch number: 418160001); Mats L Classic (MLC: bacterial thermostable α-amylase, standardised activity ≥7400 TAU/g of the preparation, batch number: 18001050), Mycolase LV (MLV: fungal α-amylase, standardised activity ≥2250 FAU/g of the preparation, batch number: 8192120011) and Brewers Compass (BC: bacterial α-amylase, bacterial β-glucanase, fungal cellulase and bacterial endopeptidase, standardised activity ≥7500 RAU/g of the preparation, batch number: 4192072011). Enzyme preparations were added to the studied mashes at the start of congress mashing at the highest dosage recommended by the producer, which equalled 200 mg of FBR for 1 kg of malt; 600 mg of FNL for 1 kg of malt; 1 g of MAX for 1 kg of malt; 275 mg of MLC for 1 kg of malt; 40 mg of MLV for 1 L of wort; 4 g of BC for 1 kg of malt; and 1.2 mL of AM for 1 kg of malt. Methods of assessment of the enzyme activity, as well as enzyme activity units are described in the Supplementary Materials.

### 2.5. Malting Procedure

#### 2.5.1. Steeping and Germination

**Figure 1:**
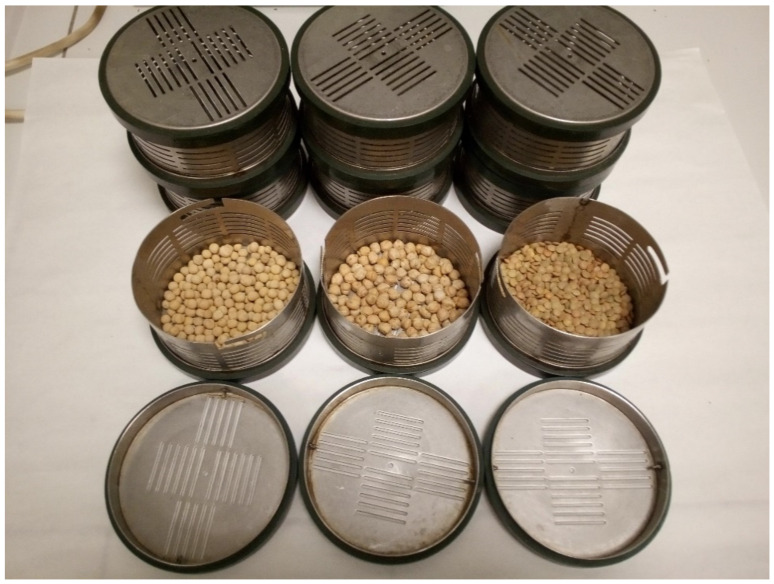
Malting containers with unmalted legume seeds: soy, chickpea, and lentil (from the left to right).

Water content in legume seeds was analysed by the Brabender MT moisture analyser (Brabender GmbH & Co, Duisburg, Germany). A total of 50 g portions of seeds were weighed and measured into stainless steel malting containers from the Automatic Micro-Malting System (Phoenix Systems, Adelaide, Australia) (Figure 1). Weight of the malting kit (container and grain) were weighed. The changes in moisture content of the legume seed samples were assessed by the changing weight of the malting kit. Steeping was executed by submerging containers in tap water (15 °C) for 5 h, removing containers from water and keeping them in humid air in a refrigerated malting cabinet (90–95% relative humidity, 15 °C) for 19 h, submerging containers in fresh tap water (15 °C) for 5 h and performing the last air rest in humid air (90–95% relative humidity, 15 °C). After 48 h of steeping, seeds acquired moisture content over 45%. After removing all unabsorbed water from seeds, the process of germination began. Grain was kept in the same malting containers in a refrigerated malting cabinet (90–95% relative humidity, 15 °C) and germinated for 144 h. During the germination process, germinated grains were mixed once every 24 h to avoid rootlets entanglement. Deficiencies in the mass of the malting sets, resulting mainly from water evaporation, were supplemented by adding distilled water, in order to maintain constant humidity of seeds throughout germination.

#### 2.5.2. Kilning and Grinding

Kilning (23 h) was performed immediately after 144 h of germination. Malting containers were loaded into a UF110 Plus dryer (Memmert GmbH + Co, Schwabach, Germany) and kilned in the following conditions: 50 °C (18 h and 50 min), ramp up to 65 °C (10 min), 65 °C (2 h and 50 min), ramp up to 82 °C (10 min), 82 °C (2 h). After kilning, malt was transferred into tightly closed containers, which prevented moisture absorption during the cooling period. After temperature of malt dropped to 25 °C, rootlets of the malt were manually removed and malt was grinded on the Bühler Miag disc mill DLFU (Bühler, Uzwil, Switzerland), according to the Analytica EBC (European Brewery Concention) 1.1 method [16]. Gradation tests were not performed, because it was assumed that differences in legume malt grinding (compared to typical malt grinding) should be a subject for another study.

### 2.6. Mashing—Congress Wort Production

#### 2.6.1. Congress Wort Production from 100% Legume Malt

**Figure 2:**
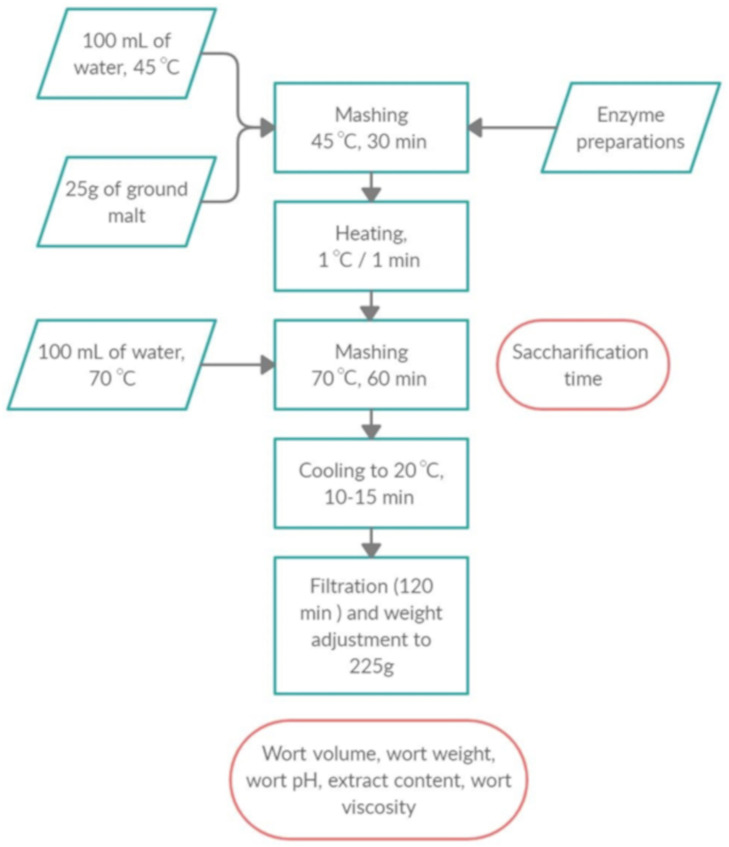
Mashing procedure for 100% legume seed malts.

Congress worts were produced in the automated laboratory mashing machine (LB Electronic, Lochner Labor + Technik, Berching, Germany) according to the Analytica EBC method 4.5.1, with modifications, due to the lower weight of grain samples [17]. Filtered wort was collected for analyses. Legume malt congress worts were prepared in duplicate. Mashing procedure is shown in the Figure 2. Mashes were prepared from chickpea seed malt, lentil seed malt, pea seed malt, and vetch seed malt, with and without external enzymes (FBR, FNL, MAX, MLC, MLV, BC, and AM). Wort prepared from Pilsen malt (M) was the control sample.

#### 2.6.2. Congress Wort Production with Addition of 30% Gelatinised Legume Malt

**Figure 3:**
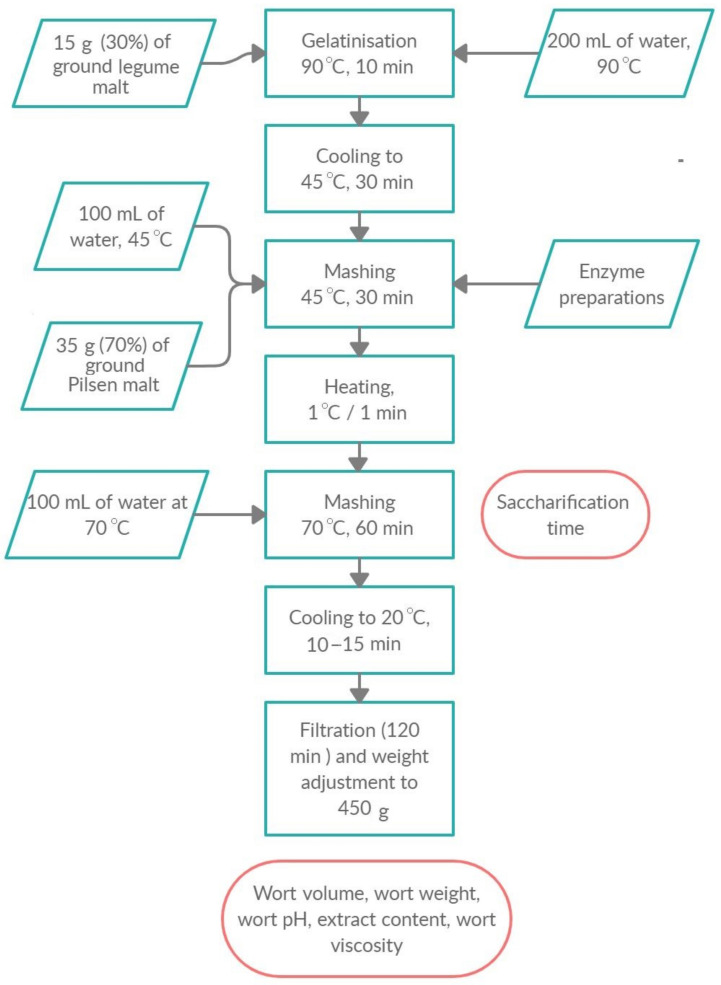
Mashing procedure for mashes with 30% gelatinised legume seed malts.

Analysis has been carried according to the Analytica EBC 4.5.1 method with modifications and is shown in the Figure 3 [17]. After the filtration process, filtered wort has been collected for analyses. Congress worts made with addition of 30% gelatinised legume malt have been prepared in duplicate. Mashes have been prepared from chickpea malt, lentil malt, and vetch malt with and without use of external enzymes (MAX). Control sample has been M2, where 30% of gelatinised legume seed malt has been substituted with 30% of Pilsen malt that also underwent gelatinisation. Congress wort with 30% of gelatinised pea malt has not been produced due to the lack of available plant material.

### 2.7. Analyses of the Acquired Worts

All of the worts were assessed by the same methods.

#### 2.7.1. Saccharification Time

Saccharification time was assessed by the Analytica EBC 4.5.1 method. After adding water at a temperature of 70 °C to the mashes, measurement of saccharification time started, and was performed in 5 min intervals. If the iodine solution did not turn the mash blue, it meant that full saccharification of the starch occurred, and value of saccharification time was noted. The last test was performed after 60 min of mashing at a temperature of 70 °C.

#### 2.7.2. Wort pH

Wort pH was assessed using pH-meter (MP220, Mettler Toledo, Columbus, OH, USA) in worts collected after the filtration process. Temperature of the tested sample was equal to 20 °C. Measurement was performed in duplicate for each wort sample.

#### 2.7.3. Wort Extract Content

Extract content of the worts was assessed with the use of densimeter (DMA 35, Anton Paar, Graz, Austria) in filtered congress wort with temperature adjusted to 20 °C. Measurements were performed in duplicate for each wort sample.

#### 2.7.4. Wort Volume

Wort volume was recorded from the scale of the graduated cylinder after the filtration process.

#### 2.7.5. Wort Viscosity

Wort viscosity was assessed according to the Analytica EBC 8.4 method with the use of falling ball KF 10 viscometer (Rheotec Company, Schorisse, Belgium) [18]. Temperature of the wort was adjusted to 20 °C prior to analysis. In the cases of some wort samples, the acquired volume of the wort was too low to perform a viscosity test. Measurement was performed in duplicate for each tested wort sample.

#### 2.7.6. Simplified Brewhouse Efficiency

Brewhouse efficiency is a parameter, which tells how much of the substances present in malt were transferred into the wort as a result of mashing [19]. Simplified brewhouse efficiency of the worts was calculated based on the wort volume, density of the wort, extract content in the wort, and mass of the malt, according to the formula below [20]:

BE = E ∙ 10 ∙ (V_k_/V_max_) (1)

BE—simplified brewhouse efficiency (%);
E—extract content of the wort (°Plato);
V_k_—final volume of the wort (mL);
V_k_—maximal volume of the wort—for 2.6.1 set at 200 mL, for 2.6.2 set at 400 mL.

### 2.8. Analyses of Phenolic Components and Antioxidant Activity of Legume Seed Malts and Legume Seed Malt Worts

#### 2.8.1. Preparation of the Worts

Worts, prior to the analysis of the concentration of phenolic compounds, as well as their antioxidant activity, were centrifuged (10 min, 5000 rpm) in the laboratory centrifuge MPW-351R (Warsaw, Poland) and filtered through the paper filter. Only chickpea malt wort (C), lentil malt wort (L), pea malt wort (P), vetch malt wort (V), Pilsen malt wort (M), gelatinised chickpea wort (GC30), gelatinised lentil wort (GL30), and gelatinised vetch malt wort (GV30), due to the lack of stored samples, were assessed.

#### 2.8.2. Preparation of the Methanol Extracts from Legume Seeds, Legume Seed Malts, and Barley Malt

To assess concentration of phenolic compounds and antioxidant activity of the legume seed malts, as well as control Pilsen malt, methanol extracts were produced, according to the modified method of Nowak [21]. A total of 20 g of Pilsen malt, legume seed malt, and legume seed samples were finely ground in a laboratory mill WZ-1 (Bydgoszcz, Poland). A total of 2.5 g of ground malt/seed samples were weighed and transferred to 50 mL polypropylene falcon tubes. A total of 40 mL of 80% (*v*/*v*) methanol was added to the falcon tubes. Samples were sonicated in a XUB5 ultrasonic bath XUB5 (Shepreth, Great Britain) for 15 min and left in a fridge at 6 °C for 12 h. After 12 h, falcon tubes were sonicated again for 15 min and centrifuged at 6000 rpm for 10 min. Only the top, clear part of the extract, without any sediment particles, was analysed.

#### 2.8.3. Concentration of Phenolic Compounds

Total content of polyphenol compounds in worts was determined using spectrophotometric Folin–Ciocalteu (F–C) method [22]. A total of 0.1 mL of wort/methanol extracts, followed by 0.2 mL of F–C reagent were pipetted into polystyrene cuvettes. After 3 min, 1 mL of 20% (*v*/*v*) sodium carbonate solution in water and 2 mL of distilled water were added into cuvettes, which were then stored in a dark place. After 1 h, absorbance of the prepared samples was analysed using A560 spectrophotometer (AOE instruments, Shanghai, China) with the wavelength set at 765 nm. Distilled water was used as a blind sample. Results were presented as an average value from three measurements for worts and as an average from nine measurements for legume seed malts/legume seed extracts. Results were expressed as mg of gallic acid equivalents per 100 mL in the case of worts, and as mg of gallic acid equivalent per 100 g of seed or malt in the case of legume seeds and legume seed malts. Calibration curve in the range of 10–200 mg gallic acid equivalent (GAE)/100 mL was used to read the results.

#### 2.8.4. ABTS•+ Assay

Antioxidative ability of the tested samples was assessed by means of the ABTS•+ assay [23]. Samples of wort or methanol extracts (0.03 mL) were mixed in a polystyrene cuvette with 3 mL of ABTS+• water solution. Absorbance of the ABTS+• solution equalled 0.700 at the wavelength of 734 nm. After 6 min, the absorbance of the tested samples was measured. Nine measurements were performed for methanol extracts and three for the worts. Results were expressed as µmol Trolox equivalent (TE) of antioxidative capacity per 1 mL of the wort (µmol TE/mL) or µmol Trolox equivalent (TE) of antioxidative capacity per 1 g of legume seed/legume seed malt. Distilled water was used as a blank sample.

#### 2.8.5. DPPH• Assay

Another method to assess antioxidative abilities of malts and worts was the DPPH• assay [24]. Samples of wort or methanol extracts (0.1 mL) were mixed with 2 mL of 0.04 mmol/L DPPH• solution in ethanol and 0.4 mL of distilled water in a polystyrene cuvette. After 10 min of incubation at room temperature, the absorbance was measured with a spectrophotometer at the wavelength of 517 nm. The data were expressed as Trolox equivalent (TE) of antioxidative capacity per 1 mL of the wort or 1 g of legume seed/legume seed malt (mmol TE/mL or mmol TE/g). All measurements were performed in triplicate for worts and in nine repetitions for seed/malt extracts. Ethanol was used as a blank sample.

#### 2.8.6. FRAP Assay

In the FRAP assay, capacity of the methanol extracts or worts to reduce iron from ferric 2,4,6-tris(2-pyridyl)-1,3,5-triazine (Fe (III)-TPTZ) was assessed [25]. Reagent was prepared by mixing 10 mmol 2,4,6-tris(2-pyridyl)-1,3,5-triazine (TPTZ)/L reagent with 20 mmol/L ferric (III) chloride in acetate buffer (pH 3.6). 0.1 mL of wort/methanol extract was mixed in polystyrene cuvette with 0.9 mL distilled water and 3 mL of ferric complex. Change in absorbance was measured after 10 min. Quantitative analyses were performed by the external standard method using ferrous (II) sulphate (0.2 mmol/L) as the reference standard. Absorbance measured at wavelength of 593 nm was correlated with the concentration of the ferrous (II) sulphate. Three measurements were performed for each of the analysed worts and nine measurements were performed in the case of methanol extracts.

### 2.9. Statistical Analysis

The results acquired in this work were statistically analysed using Statistica 13.5 (StatSoft, Tulsa, OK, USA) using one-way ANOVA (α = 0.05). Duncan’s test was used to calculate differences and assess homogenous groups between the means.

## 3. Results

### 3.1. Analyses of Physicochemical Parameters of the Congress Worts Produced from Legume Seed Malts

Results acquired from the analyses of the physicochemical parameters of the worts are shown in Table 1, Table 2, Table 3, Table 4 and Table 5.

#### 3.1.1. Saccharification Time of the Worts

Mash prepared from Pilsen malt (M) saccharified in the first 10 min of mashing at temperature of 70 °C. Most of the mashes prepared from 100% legume seed malts were characterised with worse saccharification time. Only V-MAX and L-MLV saccharified in the same time, as M. From the legume seed malt mashes, vetch malt was saccharified in most of the mashing trials (V, V-MAX, V-MLC, V-BC, V-AM), while V-MAX acquired the fastest saccharification time (10 min) and V-AM the slowest (30 min). Chickpea malt mashes were not saccharified in samples with and without enzyme preparations. Lentil malts were not saccharified in a test without addition of external enzymes, although the addition of two of the enzyme preparations resulted in a saccharified lentil mash. Fastest saccharification of lentil mash was found out for L-MLV (10 min) and slowest for L-MLC (25 min). Mashes made out of pea malt also saccharified only in two samples with addition of enzymes. Slowest saccharification time was found out for P-AM (40 min) and the fastest for P-MLC (25 min). All mashes prepared with the addition of 30% gelatinised legume seed malt saccharified in the first 10 min, with and without the addition of Maxazyme enzyme preparation.

#### 3.1.2. pH of the Worts

pH of the worts prepared from legume seed malts was higher than pH for M (5.33). The type of the legume seed malts had significant impact on pH of acquired worts. P had highest pH value (5.7), V and L were characterised by similar pH (5.53 and 5.51) and pH of C was lowest (5.44). The addition of FBR increased pH of all the worts. Similar effects could be noted for worts prepared with the addition of MAX, MLV, and AM. FNL lowered pH of the V-FNL sample (from 5.53 to 5.4). The addition of MLC lowered pH of V-MLC (5.11), P-MLC (5.34), and L-MLC (5.25), but slightly increased it in the case of C-MLC (5.51). BC had little influence on the pH of the legume seed malt worts, increasing it by 0.04–0.07 for P-BC, L-BC, and C-BC and lowering it by 0.06 for V-BC. The type of the legume seed malt used in the samples with 30% gelatinised legume seed malt had a small impact on the wort pH and the addition of MAX did not change pH of the wort significantly.

#### 3.1.3. Extract of the Worts

In comparison with the samples prepared from the legume seed malts, M was always characterised with the highest extract content. Extract content in L was the lowest from all of the tested samples (1.59 °Plato). In case of lentil malt worts, increase of the extract content due to the activity of enzyme preparations ranged from 0.74° to 1.59 °Plato (2.33° for L-MAX to 3.4 °Plato for L-MLC). The highest extract content for the legume seed malts prepared without the addition of enzyme preparations was found for P (2.8 °Plato). Most of the used enzyme preparations did not have a significant impact on the extract content of pea malt worts. Only P-MLC was characterised with lower extract content (2.1 °Plato) than P, and P-FBR acquired higher concentration of soluble solids (3.1 °Plato). Almost all vetch malt samples with the addition of enzyme preparations were characterised with lower extract content (1.58–1.8 °Plato) than V (2.27 °Plato), with the exception of V-MLC (2.8 °Plato). Most of the enzyme preparations used on the samples made from chickpea malt slightly increased extract content in the worts (from 0.07 °Plato in C-FNL to 0.41 °Plato in C-MLV). Only in case of C-MLC (2.3 °Plato) was the extract content lower than in C (2.39 °Plato). However, there was one exceptional difference concerning the chickpea malt worts. In the sample prepared with the use of MAX, the extract content of C-MAX was highest of all from the samples prepared from 100% legume seed malts and equalled 5.51 °Plato. Gelatinisation of 30% of legume seed malt mashed with Pilsen malt resulted in mashes with extract content similar—or only slightly lower—than M2. Highest extract content was noted for GC30 (6.98 °Plato) and the lowest for GV30-MAX (6.58 °Plato).

#### 3.1.4. Volume of the Worts

Out of the mashes prepared from the legume seed malts without addition of external enzymes, the volume of the V was highest (115 mL), but it still was not as high as in M (130 mL). C acquired the lowest volume of the wort (80 mL). The addition of the FBR increased the wort volume in the case of C-FBR (115 mL) and P-FBR (130 mL). FNL also improved volume in most of the cases it was used. Volume of the chickpea, lentil, and pea mashes increased by 30–43 mL to 123 mL (C-FNL), 130 mL (L-FNL), and 145 mL (P-FNL). Both Filtrase enzymes reduced volume of vetch malt mashes to 90 mL (V-FBR) and 70 mL (V-FNL). The addition of MAX increased volume of the pea, lentil, and chickpea seed mashes by 15–25 mL to 130 mL (P-MAX), 125 mL (L-MAX), and 95 mL (C-MAX). Similar results were found for mashes prepared with the addition of MLC, which also allowed for acquiring higher wort volumes for P-MLC (150 mL), L-MLC (140 mL), and C-MLC (95 mL). The addition of MLV improved wort volume for P-MLV (135 mL), V-MLV (125 mL), and L-MLV (130 mL), but drastically reduced volume of C-MLV (35 mL). BC, a composition of many enzymes, increased volume of all the worts. AM increased volume of the P-AM (135 mL) and C-AM (110 mL), but did not affect volume of the worts from vetch or lentil malts. Volume of the worts prepared with the gelatinised legume seed malts should not be compared to the legume seed malt samples, because they were prepared from the greater amount of malt (50 g) and water (400 mL). Nevertheless, it is interesting to note that the volume of the worts acquired from GL30 and GL30-MAX (215 and 205 mL) were not much higher than the volume of the P-MLC (150 mL), which was made from 25 g of malt and 200 mL of water. The highest volume of wort from samples prepared with gelatinised legume seed malts was acquired from GV30-MAX, GC30, and GV30 (280, 270 and 270 mL, respectively). Interestingly, the addition of MAX did not significantly improve wort volume in the samples prepared with 30% of legume seed malt.

#### 3.1.5. Viscosity of the Worts

Wort viscosity could not be assessed in the case of many samples, because volume of some collected worts was not high enough to perform analysis in the KF 10 Viscometer. In the samples prepared from legume malts, control sample M was characterised with the highest viscosity (1.75 mPa∙s). The addition of enzyme preparations (FBR, FNL, MAX, MLC, MLV, BC) reduced viscosity of the worts prepared from legume seed malts. P acquired the highest viscosity (1.5 mPa∙s), which was reduced in the greatest amount in P-AM to 1.31 mPa∙s. Viscosity of V equalled 1.63 mPa∙s and was significantly lowered to 1.09 mPa∙s for V-MLC. Viscosity of L and C was not analysed, although tests could be performed for some of the samples produced from these malts with the use of external enzymes. The lowest viscosity in the case of lentil malt was found for L-AM (1.29 mPa∙s) and the highest for L-MLC (1.51 mPa∙s). Viscosity of chickpea seed malt worts could be analysed only in four samples, ranging from 1.35 mPa∙s (C-FNL) to 1.39 mPa∙s (C-FBR). Viscosity was tested in all of the samples prepared with the addition of 30% gelatinised legume malts and ranged from 1.54 mPa∙s for GL30-MAX to 1.62 mPa∙s for GV30 and GC30.

#### 3.1.6. Simplified Brewhouse Efficiency

Brewhouse efficiency is a parameter that can more precisely tell how malt is modified compared to the sole analysis of wort extract or volume of the acquired wort [19]. Worts made out of legume malts without the addition of enzyme preparations were characterised by a poor brewhouse efficiency. The lowest was acquired by L (6.4%) and the highest by P (12.49%). The addition of external enzymes improved brewhouse efficiency in the case of pea, lentil, and chickpea malts. In the case of samples prepared with pea malts, the lowest increase of efficiency was noted for P-MLC (12.73%) and the highest for P-FBR (16.27%). Brewhouse efficiency was three times higher for L-MLC (19.29%) than for L, and was one of the highest efficiencies recorded for pure legume seed malt samples in this study. Worts made with the use of 30% gelatinised legume seed malt were characterised by far better brewhouse efficiency than pure legume seed malt worts. The highest efficiency was acquired by GC30 (77.52%), as well as by GV30 and GV30-MAX (74.42% and 75.65%). Interestingly, the addition of MAX did not result in improved brewhouse efficiency in these samples.

### 3.2. Analyses of the Phenolic Components and Antioxidant Activity of Legume Seed Malts and Legume Seed Malt Worts

#### 3.2.1. Concentration of Phenolic Components and Antioxidant Activity of the Legume Seeds, Legume Seed Malts, and in Barley Malt

Results of the F–C analysis and ABTS^+•^, DPPH^•^, and FRAP assays performed on the legume seeds prior to the malting process, legume seed malts, and barley malt, are shown in Table 6.

Malting increased concentration of phenolic compounds in all of the legume seeds tested in the study. The greatest, with an almost 3-fold increase, was noted for chickpea seeds (CS) and chickpea seed malt (CSM) (from 44.09 mg GAE/100 g to 112.14 mg GAE/100 g). The lowest increase in concentration of phenolic compounds was found out for VS and VSM (from 50.33 mg GAE/100 g to 61.04 mg GAE/100 g). It is worth noting that VS was characterised by the highest concentration of phenolic compounds for unmalted seeds and by the lowest concentration for malted seeds. Barley malt was characterised with greater concentration of phenolic compounds than all the legume seed malts.

#### 3.2.2. Concentration of the Phenolic Components and Antioxidant Activity in Legume Seed Malt Worts and Worts Prepared from Gelatinised Legume Seed Malts

F–C analysis of the phenolic compounds, as well as the ABTS^+•^, DPPH^•^, and FRAP assays were performed only for selected worts due to the lack of research material. Results of these analyses are shown in Table 7.

M was characterised by the highest antioxidant activity from the tested worts, however, in three tested worts (V, GC30, GV30), greater concentration of phenolic compounds was detected. V was characterised by the highest concentration of phenolic compounds (38.48 mg/100 mL) out of the worts prepared with the use of legume seed malts and acquired the highest antioxidant activity out of the four legume seed malt wort samples. In L, concentration of phenolic compounds was the lowest and equalled 33% of the amount detected in V, similar as C (which possessed slightly higher concentration of phenolic compounds than L, 36% of these detected in V). It is interesting to note that, in the GC30, GL30 and GV30 these differences were not as exceptional as in 100% legume seed malts. Further attention should be focused on the concentration of phenolic compounds in GC30, which, as mentioned before, was higher than M, despite C possessing lower concentration of phenolic compounds than M.

## 4. Discussion

### 4.1. Analyses of Physicochemical Parameters of the Congress Worts Produced from Legume Seed Malts

#### 4.1.1. Saccharification Time

Saccharification of the mashes is a result of hydrolysis of starch by the amylolytic enzymes present in the mash. For the full saccharification of the mash to happen, firstly, starch in the plant material must undergo a process of gelatinisation. Only then can it be hydrolysed by the amylolytic enzymes, such as α-amylase or β-amylase. Unfortunately, starch present in the legume seeds has higher gelatinisation temperature than starches present in the typically malted grains, such as barley or wheat [26]. However, the malting process can reduce gelatinisation temperature of starches even by 20 °C, depending on the malting conditions [27]. Currently there is no knowledge about characteristics of starch granules present in the legume seeds malts and about optimal temperature for legume seed malt enzymes to complete saccharification of starch present in legume seed malt mashes. There are many factors that can hinder starch hydrolysis, such as lack of amylolytic enzymes [28], non-catalytic binding of enzymes on non-substrate polymers, or physical barriers preventing access to the starch [6]. The conducted study shows that in the case of lentil malt, the main reason seems to be a lack of amylolytic enzymes, because samples L-MLC and L-MLV, prepared with external α-amylases, saccharified fully. Interestingly, L-AM (mash with addition of amyloglucosidase) did not undergo full saccharification. However, P-AM and P-MLC saccharified fully, but hydrolysis of starch was not complete in the case of P-MLV. Mashes prepared from chickpea malt were not saccharified, despite the addition of different compositions of external enzymes, which might show that the lack of internal enzymes is not the main problem in processing chickpea malt. The addition of proteolytic enzymes, as well as cellulases and β-glucanases did not improve saccharification of chickpea malt, so the possibility of starch being blocked by proteins, cellulose, or β-glucanase might also be excluded in the case of chickpea malt. Analysis of vetch malt mashes is one of the most interesting instances in this study, because V saccharified fully, but the addition of external enzymes mostly hindered saccharification of this malt. It is possible that used enzymes released some substances from vetch malt, which prevented proper activity of amylases, but the conducted study did not possess adequate means to confirm this assumption. In the case of the mashes prepared with the addition of gelatinised legume seed malts, all of the samples with 30% of legume malt saccharified fully. It might show, especially in the case of the chickpea malt, which gelatinisation temperature of the starch present in the legume malts is higher than 70 °C, and 70% of Pilsen malt addition possess a sufficient amount of enzymes to hydrolyse starch in the 30% of legume malt.

#### 4.1.2. Wort pH

Analysis of the worts prepared by the congress mashing shows how much pH of the congress worts made from legume seed malts deviates from optimal pH for enzymes present in the barley malt (5.6–5.8 pH for α-amylase, 5.4–5.5 pH for β-amylase) [29]. These parameters might be optimal for the enzymes present in the legume seed malts; however, optimal pH of legume seed malt enzymes is currently unknown. In the prepared mashes, pH was not modified, so the wort pH is a result of all the substances, which were extracted from the malts and introduced to the worts. pH is an important parameter in the wort production, because it is one of the factors regulating activity of the external and internal enzymes present in the mash [29]. Most of the legume seed malt worts acquired lower pH value than non-typical worts produced from 100% oat malt in a study conducted by Klose et al., in which pH of the oat worts ranged from 5.9 to 5.99 [30]. pH of most of the legume malt worts was also lower than pH of rice malt wort acquired during congress mashing in the study conducted by Mayer et al. [31]. Beer with the addition of unmalted wheat and corn grist, produced and analysed by Vinko Krstanović et al., resulted in wort of pH higher (5.73) than most of the legume seed malt worts [32]. Wort pH is, usually, in the traditional congress mash analysis, a useful predictor for the extract of the acquired wort, as lower pH is known to improve saccharification, extract content, as well as filtering time [33]. However, as was said earlier, there is no available research concerning optimal pH values for activity of enzymes present in the legume seed malts. Correlation between wort pH and saccharification time could not be found in the case of legume malts used in this study and certain conclusions about the impact of the legume seed malts on the wort pH could not be acquired from the performed study. In the samples without the addition of external enzymes, samples prepared from legume seed malts were characterised with higher pH. This is confirmed by the samples prepared with the use of gelatinised legume seed malts: all samples with 70% of Pilsen malt acquired slightly higher pH value than M2. Without knowledge about optimal pH of the legume malt enzymes, it is hard to assess whether change in pH is positive or negative. The only certain result acquired from the wort pH analysis is that, with the increase in the amount of legume seed malt added to mash prepared with Pilsen malt, the activity of barley enzymes will decrease; this could create difficulties in acquiring wort with acceptable qualities.

#### 4.1.3. Wort Extract

Extract of the collected worts is one of the parameters, which, combined with the volume of the wort, can tell how much of the substances can be transferred from the malt (brewhouse efficiency). In congress wort analysis, the best results are obtained when large volume of wort with high extract content is acquired; however, acquiring wort with perfect parameters is rarely the case in samples prepared from special, non-typical malts [11]. The suboptimal worts, either characterised with low extract content and high volume, or with high extract content and low volume, possess some flaws. Usually, with rise in the wort extract, wort viscosity increases, which is a hindrance in many of the processes [29]. Worts with low extract content are typically characterised by lower viscosity; however, for use in many branches of industry, such as production of malt extract or dietary supplements, beer brewing, baking industry, or production of nutrients for microorganisms, a product with high concentration of dissolved solids is preferred [34,35,36]. Production of the concentrated malt products from worts with low extract content require more water to be removed. Water is a liquid with very high thermal capacity, so water evaporation needs a great amount of energy to be utilised; thus, increasing costs of the technological process [37]. The extract content of worts prepared from the legume seed malts in most samples was far lower than extract content in M or M2. In nearly all of the samples in which enzyme preparations were used, worts made from vetch malt were characterised with the lowest extract content, which might explain the presence of the hard-to-digest seed cover characteristic for vetch, as well as the higher content of non-nutritional factors than in other legumes [38,39]. Extract content in the worts prepared only from the 30% gelatinised legume seed malt reached similar levels as the control samples, prepared from Pilsen malt. The most promising results were found for the C-MAX sample, which, for 100% legume seed malt wort, acquired astonishingly high extract concentration, which might indicate that proteins are the main substances present in the chickpea malt, which hinder its extractability. Similar factors could not be noted for any of the other 100% legume seed malt samples, although analyses of brewhouse efficiency, discussed later, can also help in identifying critical impediments in the proper extraction processes.

#### 4.1.4. Wort Volume

Low wort volume combined with low extract of collected wort show that the malt sample has very poor extractability. Disadvantages of worts with low extract content and high volume, as well as worts with high extract content and low volume, were discussed in Section 4.1.3. Filterability is an important parameter in the wort production, because high concentration of substances, which can hinder filtration, will lengthen the process of wort production. Factors affecting wort filtration are concentration of soluble substances in the wort, wort viscosity, concentration of phenols, content of insoluble polysaccharides, and characteristics of the grain/seed bed, through which the wort is filtered [29,40]. The worts prepared from the legume seed malts were characterised with lower wort volume than worts acquired from barley malt. The addition of the external enzymes to the prepared legume worts often improved wort filtration, which, due to the specificity of enzymes, might help in characterisation, where substances present in the special malts may cause difficulties. Volume of the worts acquired from the gelatinised legume seed malt samples was acceptable in the case of GV30, GC30, GV30-MAX, and GC30-MAX.

#### 4.1.5. Wort Viscosity

According to Kunze, viscosity of the wort acquired from typical Pilsen malt should fall into the range of 1.5 mPa∙s to 1.6 mPa∙s [29]. In the conducted study, only three of the worts (GL30, GV30-MAX, GL30-MAX) were characterised by these parameters. Almost all of the legume malt worts in which wort viscosity could be assessed (with the exception of V) were characterised by lower viscosity, which was probably a result of poor malt extractability, because viscosity of the wort usually increases with the increase in the extract content [41]. This is confirmed by the results acquired in this study: most of the worts with high extract concentration acquired highest values for wort viscosity. Only in one of the samples, V, was wort with high viscosity characterised with low wort extract. This might indicate a possibility of extracting some substances, which, despite being present only in small concentrations in the wort, substantially increase wort viscosity.

#### 4.1.6. Brewhouse Efficiency

Brewhouse efficiency is a parameter that takes into consideration both wort volume as the extract content [19]. The goal of the typical mashing process is to transfer most of the substances present in the mash to the filtered wort [42]. Brewhouse efficiency shows that legume malts, created by the malting features used typically for barley, are poorly modified. Nevertheless, samples prepared with the addition of 30% gelatinised vetch and chickpea malt show rather good efficiency, which means that they could be possibly used in the brewing technology in a way presented in this paper. However, more studies need to be performed on the quality and composition of the acquired legume seed malt wort, because legumes are rich in anti-nutritional factors, such as lectins, phytic acid, enzyme inhibitors, saponins, and haemagglutinins. Currently, it is not known whether they transfer easily to the wort acquired from legume seed malt [43]. However, it is known that some of the processes used in creating malt and wort, such as soaking, germination, thermal processing, and milling, reduce concentration of many of these harmful substances [44,45,46]. Future studies might show that low extractability of the legume seed malt will we be its advantage, because it might leave anti-nutritional substances in the spent legume seeds. Of course, the opposite might also be true—wort might be full of anti-nutritional factors, which would make spent legume seeds a far more interesting product, reduced by its disadvantageous substances.

### 4.2. Analyses of the Phenolic Components and Antioxidant Activity of Legume Seed Malts and Legume Seed Malt Worts

Germination of seeds and grains, which is one of the main steps in the production of malt, has been analysed by many researchers in the past and it is commonly known that sprouted seeds possess high nutritional and antioxidative properties, and are eaten as a health food all around the world [47,48]. Many researchers also confirm that malt created from the grain possesses higher concentration of phenolic compounds, as well as higher antioxidant activity than unmodified grain [11,29]. It is therefore not surprising to see that legume seed malts contain higher concentrations of phenolic components and higher antioxidant activities than their unmalted counterparts. The most interesting fact about the results acquired in this study is the surprisingly high antioxidant activity of the VS and VSM, which possessed far smaller concentration of phenolic components than BM. However, it might be explained by the fact that legume seeds, especially these possessing dark, hard cover, as *Vicia sativa*, contain flavonoids and condensed tannins, which may increase antioxidant activity in a greater extent than phenolic compounds present in the BM [49]. It is also important to note that, in the case of legume seed malts, high concentration of phenolic compounds in malt did not always result in high concentration of phenolic compounds in the wort produced from this malt. From legume seed malts, VSM possessed the lowest concentration of these chemicals, but V acquired concentration of phenolic components, as well as antioxidant activity, second only to the M. Antioxidant activity of legume seed malts also did not have a straightforward impact on the properties of the legume seed malt worts. CSM was characterised with higher antioxidant activity (ABTS^+•^ assay) than LSM and similar antioxidant activity as PSM, but the same parameter of L and S was greater than of C. This results show that extraction of bioactive substances from legume seed malts is more difficult that extraction of the substances from the typical barley malt, so novel malting or mashing procedures need to be applied to the legume seeds and malts produced from them. Without detailed studies about composition of phenolic compounds in legume seed malts and worts prepared from these malts, it is hard to draw conclusions solely from the Folin–Ciocalteu, ABTS^+•^, DPPH^•^, or FRAP analysis. It is only certain that substitution of barley malt with gelatinised legume seed malts might increase concentration of phenolic compounds in the wort (in the case of GC30 and GV30); however, it decreases its antioxidant properties. In the study conducted by Gąsior et al., it was shown that use of traditional beer brewing adjuncts, such as chocolate malts or roasted, unmalted barley, increased concentration of phenolic compounds as well as antioxidative properties of the worts to a greater extent than the addition of gelatinised legume seed malts conducted in this research [15].

## 5. Conclusions

The conducted study shows that production of malts from legume seeds is possible, but technological properties of acquired malts are inferior to typical barley malts. Legume seed malt worts produced in this study by congress mashing were characterised with lower extract content, lower wort volume, and lower brewing efficiency than typical worts produced from barley malt. However, the addition of enzyme preparations to the mashes improved some of the characteristics of legume seed malt worts. Wort samples created from 30% addition of gelatinised vetch malt or chickpea malt were characterised with sufficiently good properties, which shows that legume malts might be used in the future as a substitute of unmalted adjuncts. The study also showed that malting increased concentration of phenolic compounds and antioxidant activity of the legume seeds; however, traditional mashing conditions only allowed producing legume seed malt worts with lower antioxidant activity than wort produced from barley malt. More research is needed on the composition of worts made from legume seed malts and on the influence of different malting conditions on the properties of legume seed malt worts.

## Figures and Tables

**Table 1:** Physicochemical parameters of chickpea malt worts with and without addition of enzyme preparations.

Sample ^1^	Saccharification Time ^2^	pH	Extract (°Plato)	Wort Volume (mL)	Wort Viscosity (mPa∙s)	Brewhouse Efficiency (%)
C	X	5.44 ± 0.03 ^cd^	2.39 ± 0.11 ^ef^	80 ± 5 ^d^	n.d.	9.53 ± 0.16 ^d^
C-FBR	X	6.15 ± 0.07 ^a^	2.60 ± 0.05 ^d^	115 ± 5 ^b^	1.39 ± 0.06 ^b^	14.97 ± 0.94 ^c^
C-FNL	X	5.55 ± 0.07 ^bc^	2.46 ± 0.04 ^def^	122.5 ± 2.5 ^ab^	1.35 ± 0.07 ^b^	15.07 ± 0.07 ^c^
C-MAX	X	5.50 ± 0.04 ^bc^	5.51 ± 0.03 ^b^	95 ± 5 ^c^	n.d.	26.18 ± 1.52 ^b^
C-MLC	X	5.51 ± 0.02 ^bc^	2.30 ± 0.06 ^f^	95 ± 5 ^c^	1.39 ± 0.03 ^b^	10.94 ± 0.86 ^d^
C-MLV	X	5.63 ± 0.02 ^b^	2.80 ± 0.02 ^c^	35 ^e^	n.d.	4.91 ± 0.04 ^e^
C-BC	X	5.51 ± 0.03 ^bc^	2.54 ± 0.03 ^de^	110 ± 5 ^b^	1.37 ± 0.02 ^b^	13.98 ± 0.80 ^c^
C-AM	X	5.51 ± 0.01 ^bc^	2.53 ± 0.02 ^de^	110 ± 5 ^b^	1.37 ± 0.03 ^b^	13.95 ± 0.75 ^c^
M	10	5.31 ± 0.05 ^d^	6.84 ± 0.07 ^a^	130 ^a^	1.75 ± 0.03 ^a^	44.47 ± 0.46 ^a^

^1^ C—wort from chickpea seed malt, C-FBR—wort from chickpea malt mashed with addition of Filtrase BR-X, C-FNL—wort from chickpea malt mashed with addition of Filtrase NL, C-MAX—wort from chickpea malt mashed with addition of Maxazyme NNP DS, C-MLC—wort from chickpea malt mashed with addition of Mats L Classic, C-MLV—wort from chickpea malt mashed with addition of Mycolase LV, C-BC—wort from chickpea malt mashed with addition of Brewers Compass, C-FNL—wort from chickpea malt mashed with addition of Amigase Mega, M—wort from Pilsen barley malt. Values are expressed as mean (*n* = 2) ± standard deviation in case of saccharification time, wort volume and brewhouse efficiency and as a mean (*n* = 4) ± standard deviation in case of pH, extract and viscosity. Mean values with different letters (a, b, …, f) within the same column are statistically different (*p*-value < 0.05). ^2^ “X” means that complete saccharification of the sample had not been acquired; n.d. stands for “no data” in the samples in which viscosity could not be assessed.

**Table 2:** Physicochemical parameters of lentil malt worts with and without addition of enzyme preparations.

Sample ^1^	Saccharification Time ^2^	pH	Extract (°Plato)	Wort Volume (mL)	Wort Viscosity (mPa∙s)	Brewhouse Efficiency (%)
L	X	5.51 ± 0.05 ^d^	1.59 ± 0.04 ^f^	100 ^c^	n.d.	7.95 ± 0.20 ^f^
L-FBR	X	6.12 ± 0.03 ^a^	2.60 ± 0.04 ^c^	100 ^c^	1.39 ± 0.03 ^c^	13.00 ± 0.20 ^de^
L-FNL	X	5.57 ± 0.02 ^cd^	2.41 ± 0.03 ^de^	130 ± 5 ^ab^	1.32 ± 0.01 ^cd^	15.66 ± 0.41 ^c^
L-MAX	X	5.64 ± 0.03 ^bc^	2.33 ± 0.03 ^e^	125 ± 5 ^b^	1.39 ± 0.01 ^c^	14.57 ± 0.77 ^cd^
L-MLC	25	5.25 ± 0.04 ^e^	3.4 ± 0.02 ^b^	140 ± 5 ^a^	1.51 ± 0.03 ^b^	23.80 ± 0.71 ^b^
L-MLV	10	5.6 ± 0.03 ^bcd^	2.39 ± 0.04 ^e^	130 ± 5 ^ab^	n.d.	15.55 ± 0.86 ^c^
L-BC	X	5.55 ± 0.02 ^cd^	2.4 ± 0.07 ^de^	130 ^ab^	1.37 ± 0.03 ^c^	15.61 ± 0.46 ^c^
L-AM	X	5.69 ± 0.03 ^b^	2.55 ± 0.04 ^cd^	100 ^c^	1.29 ± 0.03 ^d^	12.75 ± 0.2 ^e^
M	10	5.31 ± 0.05 ^e^	6.84 ± 0.07 ^a^	130 ^ab^	1.75 ± 0.03 ^a^	44.47 ± 0.46 ^a^

^1^ L—wort from lentil malt, L-FBR—wort from lentil malt mashed with addition of Filtrase BR-X, L-FNL—wort from lentil malt mashed with addition of Filtrase NL, L-MAX—wort from lentil malt mashed with addition of Maxazyme NNP DS, L-MLC—wort from lentil malt mashed with addition of Mats L Classic, L-MLV—wort from lentil malt mashed with addition of Mycolase LV, L-BC—wort from lentil malt mashed with addition of Brewers Compass, L-FNL—wort from lentil malt mashed with addition of Amigase Mega, M—wort from Pilsen barley malt. Values are expressed as mean (*n* = 2) ± standard deviation in case of saccharification time, wort volume and brewhouse efficiency and as a mean (*n* = 4) ± standard deviation in case of pH, extract and viscosity. Mean values with different letters (a, b, …, f) within the same column are statistically different (*p*-value < 0.05). ^2^ “X” means that complete saccharification of the sample had not been acquired; n.d. stands for “no data” in the samples in which viscosity could not be assessed.

**Table 3:** Physicochemical parameters of pea malt worts with and without addition of enzyme preparations.

Sample ^1^	Saccharification Time ^2^	pH	Extract (°Plato)	Wort Volume (mL)	Wort Viscosity (mPa∙s)	Brewhouse Efficiency (%)
P	X	5.7 ± 0.06 ^b^	2.80 ± 0.08 ^c^	110 ± 10 ^b^	1.50 ± 0.05 ^b^	15.44 ± 1.84 ^c^
P-FBR	X	6.37 ± 0.06 ^a^	3.10 ± 0.08 ^b^	130 ± 15 ^ab^	1.46 ± 0.03 ^b^	20.10 ± 1.81 ^b^
P-FNL	X	5.75 ± 0.1 ^b^	2.74 ± 0.03 ^c^	145 ± 5 ^a^	1.33 ± 0.04 ^cd^	19.86 ± 0.47 ^b^
P-MAX	X	5.86 ± 0.03 ^b^	2.76 ± 0.04 ^c^	130 ± 5 ^ab^	1.47 ± 0.03 ^b^	17.95 ± 0.95 ^bc^
P-MLC	25	5.34 ± 0.03 ^c^	2.10 ± 0.05 ^d^	150 ± 10 ^a^	1.45 ± 0.04 ^b^	15.78 ± 1.43 ^c^
P-MLV	X	5.76 ± 0.01 ^b^	2.77 ± 0.02 ^c^	135 ± 5 ^ab^	1.42 ± 0.01 ^bc^	18.7 ± 0.56 ^bc^
P-BC	X	5.77 ± 0.01 ^b^	2.92 ± 0.04 ^c^	130 ^ab^	1.43 ± 0.02 ^bc^	18.98 ± 0.26 ^bc^
P-AM	40	5.87 ± 0.02 ^b^	2.81 ± 0.03 ^c^	135 ± 5 ^ab^	1.31 ± 0.01 ^d^	18.98 ± 0.91 ^bc^
M	10	5.31 ± 0.05 ^c^	6.84 ± 0.07 ^a^	130 ^ab^	1.75 ± 0.03 ^a^	44.47 ± 0.46 ^a^

^1^ P—wort from pea malt, P-FBR—wort from pea malt mashed with addition of Filtrase BR-X, P-FNL—wort from pea malt mashed with addition of Filtrase NL, P-MAX—wort from pea malt mashed with addition of Maxazyme NNP DS, P-MLC—wort from pea malt mashed with addition of Mats L Classic, P-MLV—wort from pea malt mashed with addition of Mycolase LV, P-BC—wort from pea malt mashed with addition of Brewers Compass, P-FNL—wort from pea malt mashed with addition of Amigase Mega, M—wort from Pilsen barley malt. Values are expressed as mean (*n* = 2) ± standard deviation in case of saccharification time, wort volume and brewhouse efficiency and as a mean (*n* = 4) ± standard deviation in case of pH, extract and viscosity. Mean values with different letters (a, b, …, d) within the same column are statistically different (*p*-value < 0.05). ^2^ “X” means that complete saccharification of the sample had not been acquired.

**Table 4:** Physicochemical parameters of vetch malt worts with and without addition of enzyme preparations.

Sample ^1^	Saccharification Time ^2^	pH	Extract (°Plato)	Wort Volume (mL)	Wort Viscosity (mPa∙s)	Brewhouse Efficiency (%)
V	20	5.53 ± 0.04 ^bc^	2.40 ± 0.05 ^c^	115 ± 5 ^bc^	1.63 ± 0.03 ^b^	13.82 ± 0.89 ^b^
V-FBR	X	6.08 ± 0.07 ^a^	1.80 ± 0.06 ^d^	90 ± 5 ^e^	n.d.	8.12 ± 0.72 ^c^
V-FNL	X	5.40 ± 0.04 ^cd^	1.66 ± 0.03 ^de^	70 ^f^	n.d.	5.82 ± 0.11 ^d^
V-MAX	10	5.55 ± 0.04 ^bc^	1.71 ± 0.03 ^de^	100 ^de^	n.d.	8.56 ± 0.15 ^c^
V-MLC	25	5.11 ± 0.06 ^e^	2.80 ± 0.08 ^b^	100 ± 5 ^de^	1.09 ± 0.05 ^e^	14.02 ± 1.10 ^b^
V-MLV	X	5.57 ± 0.03 ^b^	1.60 ± 0.06 ^e^	125 ± 5 ^ab^	1.48 ± 0.03 ^c^	10.02 ± 0.78 ^c^
V-BC	20	5.47 ± 0.02 ^bc^	1.58 ± 0.03 ^e^	105 ± 5 ^cd^	1.28 ± 0.04 ^d^	8.29 ± 0.24 ^c^
V-AM	30	5.58 ± 0.02 ^b^	1.79 ± 0.01 ^d^	115 ± 5 ^bc^	1.28 ± 0.03 ^d^	10.30 ± 0.51 ^c^
M	10	5.31 ± 0.05 ^d^	6.84 ± 0.07 ^a^	130 ^a^	1.75 ± 0.03 ^a^	44.47 ± 0.46 ^a^

^1^ V—wort from vetch malt, V-FBR—wort from vetch malt mashed with addition of Filtrase BR-X, V-FNL—wort from vetch malt mashed with addition of Filtrase NL, V-MAX—wort from vetch malt mashed with addition of Maxazyme NNP DS, V-MLC—wort from vetch malt mashed with addition of Mats L Classic, V-MLV—wort from vetch malt mashed with addition of Mycolase LV, V-BC—wort from vetch malt mashed with addition of Brewers Compass, V-FNL—wort from vetch malt mashed with addition of Amigase Mega, M—wort from Pilsen barley malt. Values are expressed as mean (*n* = 2) ± standard deviation in case of saccharification time, wort volume and brewhouse efficiency and as a mean (*n* = 4) ± standard deviation in case of pH, extract and viscosity. Mean values with different letters (a, b, …, d) within the same column are statistically different (*p*-value < 0.05). ^2^ “X” means that complete saccharification of the sample had not been acquired; n.d. stands for “no data” in the samples in which viscosity could not be assessed.

**Table 5:** Physicochemical parameters of the worts prepared with addition of gelatinised legume malt with and without addition of enzyme preparations.

Sample ^1^	Saccharification Time	pH	Extract (°Plato)	Wort Volume (mL)	Wort Viscosity (mPa∙s)	Brewhouse Efficiency (%)
GC30	10	5.42 ± 0.04 ^a^	6.98 ± 0.07 ^a^	270 ± 10 ^bc^	1.62 ± 0.03 ^b^	47.14 ± 2.22 ^b^
GL30	10	5.34 ± 0.03 ^ab^	6.69 ± 0.05 ^bc^	215 ± 5 ^d^	1.58 ± 0.03 ^b^	35.96 ± 0.57 ^d^
GV30	10	5.38 ± 0.02 ^ab^	6.71 ± 0.04 ^bc^	270 ± 5 ^bc^	1.62 ± 0.02 ^b^	45.30 ± 1.11 ^bc^
GC30-MAX	10	5.37 ± 0.02 ^ab^	6.76 ± 0.04 ^b^	250 ± 5 ^c^	1.61 ± 0.01 ^b^	42.26 ± 1.10 ^c^
GL30-MAX	10	5.32 ± 0.01 ^b^	6.64 ± 0.04 ^bc^	205 ± 5 ^d^	1.54 ± 0.02 ^b^	34.03 ± 0.63 ^d^
GV30-MAX	10	5.37 ± 0.03 ^ab^	6.58 ± 0.04 ^c^	280 ± 10 ^b^	1.55 ± 0.03 ^b^	46.08 ± 1.93 ^bc^
M2	10	5.32 ± 0.03 ^b^	7.08 ± 0.04 ^a^	325 ± 5 ^a^	1.77 ± 0.03 ^a^	57.49 ± 1.17 ^a^

^1^ GC30—wort from 30% gelatinised chickpea malt and 70% barley malt, GL30—wort from 30% gelatinised lentil malt and 70% barley malt, GV30—wort from 30% gelatinised vetch malt and 70% barley malt, GC30-MAX—wort from 30% gelatinised chickpea malt and 70% barley malt mashed with addition of Maxazyme NNP DS, GL30-MAX—wort from 30% gelatinised lentil malt and 70% barley malt mashed with addition of Maxazyme NNP DS, GV30-MAX—wort from 30% gelatinised vetch malt and 70% barley malt mashed with addition of Maxazyme NNP DS, M2—wort from 30% gelatinised Pilsen barley malt and 70% barley malt. Values are expressed as mean (*n* = 2) ± standard deviation in case of saccharification time, wort volume and brewhouse efficiency and as a mean (*n* = 4) ± standard deviation in case of pH, extract and viscosity. Mean values with different letters (a, b, c) within the same column are statistically different (*p*-value < 0.05).

**Table 6:** Concentration of phenolic compounds and antioxidant activity of legume seeds, legume seed malts and barley malt.

Sample ^1^	Concentration of Phenolic Compounds (mg GAE ^2^/100 g)	ABTS^+•^ Assay (µmol TE ^3^/g)	DPPH^•^ Assay (µmol TE/g)	FRAP Assay (µmol TE/g)
CS	44.09 ± 0.60 ^f^	4.40 ± 0.04 ^g^	1.05 ± 0.03 ^f^	3.46 ± 0.02 ^g^
CSM	112.14 ± 1.29 ^b^	7.09 ± 0.06 ^d^	1.22 ± 0.03 ^e^	4.84 ± 0.02 ^e^
LS	46.50 ± 0.98 ^f^	5.66 ± 0.08 ^f^	1.61 ± 0.03 ^d^	4.77 ± 0.04 ^e^
LSM	84.81 ± 1.53 ^c^	6.06 ± 0.13 ^e^	2.12 ± 0.05 ^b^	5.78 ± 0.04 ^b^
PS	21.21 ± 1.42 ^g^	5.46 ± 0.14 ^f^	0.86 ± 0.03 ^g^	3.96 ± 0.03 ^f^
PSM	85.59 ± 1.24 ^c^	6.83 ± 0.09 ^d^	1.66 ± 0.05 ^d^	5.19 ± 0.02 ^c^
VS	50.33 ± 0.68 ^e^	9.85 ± 0.11 ^a^	1.87 ± 0.02 ^c^	4.77 ± 0.04 ^e^
VSM	61.04 ± 0.64 ^d^	8.43 ± 0.11 ^b^	1.81 ± 0.02 ^c^	4.97 ± 0.04 ^d^
BM	128.62 ± 1.27 ^a^	7.94 ± 0.10 ^c^	4.28 ± 0.04 ^a^	7.26 ± 0.04 ^a^

^1^ CS—chickpea seeds, CSM—chickpea seed malt, LS—lentil seeds, LSM—lentil seed malt, PS—pea seeds, PSM—pea seed malts, VS—vetch seeds, VSM—vetch seed malt, BM—Pilsen barley malt. Values are expressed as mean (*n* = 9) ± standard. Mean values with different letters (a, b, …, g) within the same column are statistically different (*p*-value < 0.05). ^2^ GAE— Gallic acid equivalent. ^3^ TE - Trolox equivalent.

**Table 7:** Concentration of phenolic compounds and antioxidant activity of legume seed malt worts.

Sample ^1^	Concentration of Phenolic Compounds (mg GAE ^2^/100 mL)	ABTS^+•^ Assay (µmol TE ^3^/mL)	DPPH^•^ Assay (µmol TE/mL)	FRAP Assay (µmol TE/mL)
C	13.90 ± 0.17 ^f^	0.47 ± 0.01 ^e^	0.26 ± 0.01 ^f^	0.23 ± 0.01 ^h^
L	12.70 ± 0.10 ^g^	0.58 ± 0.01 ^d^	0.37 ± 0.02 ^d^	0.35 ± 0.01 ^f^
P	16.59 ± 0.38 ^e^	0.59 ± 0.01 ^d^	0.27 ± 0.01 ^f^	0.30 ± 0.01 ^g^
V	38.48 ± 0.30 ^a^	1.21 ± 0.03 ^b^	0.92 ± 0.02 ^b^	0.71 ± 0.03 ^b^
GC30	20.82 ± 0.24 ^c^	0.61 ± 0.02 ^d^	0.42 ± 0.01 ^c^	0.43 ± 0.01 ^d^
GL30	16.31 ± 0.12 ^e^	0.62 ± 0.01 ^d^	0.31 ± 0.02 ^e^	0.39 ± 0.01 ^e^
GV30	23.01 ± 0.24 ^b^	0.74 ± 0.02 ^c^	0.38 ± 0.01 ^d^	0.52 ± 0.02 ^c^
M	19.01 ± 0.14 ^d^	1.44 ± 0.03 ^a^	1.09 ± 0.03 ^a^	1.08 ± 0.02 ^a^

^1^ C—wort from chickpea seed malt, GC30—wort from 30% gelatinised chickpea malt and 70% barley malt, GL30—wort from 30% gelatinised lentil malt and 70% barley malt, GV30—wort from 30% gelatinised vetch malt and 70% barley malt,. Values are expressed as mean (*n* = 9) ± standard. Mean values with different letters (a, b, …, h) within the same column are statistically different (*p*-value < 0.05). ^2^ GAE— Gallic acid equivalent. ^3^ TE - Trolox equivalent.

## Data Availability

We choose to exclude this statement, all the data provided is in the manuscript and Supplementary Materials.

## Supplementary Materials

The following are available online at https://www.mdpi.com/2304-8158/10/2/304/s1.
